# Association of hemoglobin A_1c_ and glycated albumin with carotid atherosclerosis in community-dwelling Japanese subjects: the Hisayama Study

**DOI:** 10.1186/s12933-015-0247-7

**Published:** 2015-06-24

**Authors:** Naoko Mukai, Toshiharu Ninomiya, Jun Hata, Yoichiro Hirakawa, Fumie Ikeda, Masayo Fukuhara, Taeko Hotta, Masafumi Koga, Udai Nakamura, Dongchon Kang, Takanari Kitazono, Yutaka Kiyohara

**Affiliations:** Center for Cohort Studies, Graduate School of Medical Sciences, Kyushu University, 3-1-1 Maidashi, Higashi-ku, Fukuoka, 812-8582 Japan; Department of Environmental Medicine, Graduate School of Medical Sciences, Kyushu University, Fukuoka, Japan; Department of Medicine and Clinical Science, Graduate School of Medical Sciences, Kyushu University, Fukuoka, Japan; Department of Clinical Chemistry and Laboratory Medicine, Kyushu University Hospital, Fukuoka, Japan; Department of Internal Medicine, Kawanishi City Hospital, Hyogo, Japan

**Keywords:** Hemoglobin A_1c_, Glycated albumin, 1,5-anhydroglucitol, Fasting plasma glucose, 2-hour postload glucose, Carotid atherosclerosis

## Abstract

**Background:**

It is not clear which glucose measure is more useful in the assessment of atherosclerosis. We investigated the associations of hemoglobin A_1c_ (HbA_1c_), glycated albumin (GA), 1,5-anhydroglucitol (1,5-AG), fasting plasma glucose (FPG), and 2-hour postload glucose (PG) with carotid intima-media thickness (IMT) in community-dwelling Japanese subjects.

**Methods:**

A total of 2702 subjects aged 40–79 years underwent a 75-g oral glucose tolerance test and measurements of HbA_1c_, GA, 1,5-AG, and carotid IMT by ultrasonography in 2007–2008. Carotid wall thickening was defined as a maximum IMT of >1.0 mm. The crude and multivariable-adjusted linear and logistic regression models were used to analyze cross-sectional associations between levels of glycemic measures and carotid IMT.

**Results:**

The crude average of the maximum IMT increased significantly with rising quartiles of HbA_1c_, GA, FPG, and 2-hour PG levels in subjects with and without glucose intolerance (GI), while no clear association was observed for 1,5-AG. After adjustment for other confounding factors, positive trends for HbA_1c_, GA, and FPG (all p for trend < 0.05), but not 2-hour PG (*p* = 0.07) remained robust in subjects with GI, but no such associations were found in those without GI. When estimating multivariable-adjusted β values for the associations of 1 SD change in glycemic measures with the maximum IMT in subjects with GI, the magnitude of the influence of HbA_1c_ (β = 0.021), GA (β = 0.024), and FPG (β = 0.024) was larger than that of 2-hour PG (β = 0.014) and 1,5-AG (β = 0.003). The multivariable-adjusted odds ratios for the presence of carotid wall thickening increased significantly with elevating HbA_1c_, GA, and FPG levels only in subjects with GI (all p for trend < 0.001). Among subjects with GI, the area under the receiver operating characteristic curve significantly increased by adding HbA_1c_ (*p* = 0.04) or GA (*p* = 0.04), but not 1,5-AG, FPG, or 2-hour PG, to the model including other cardiovascular risk factors.

**Conclusions:**

In community-dwelling Japanese subjects with GI, elevated HbA_1c_, GA, and FPG levels were significantly associated with increased carotid IMT, and HbA_1c_ and GA provided superior discrimination for carotid wall thickening compared to 1,5-AG, FPG, and 2-hour PG, suggesting that HbA_1c_ and GA are useful for assessing carotid atherosclerosis.

**Electronic supplementary material:**

The online version of this article (doi:10.1186/s12933-015-0247-7) contains supplementary material, which is available to authorized users.

## Background

It has been well established that hemoglobin A_1c_ (HbA_1c_) levels, which have been used widely as a measure of chronic hyperglycemia, are closely associated with the risk of microvascular complications, such as diabetic retinopathy, and this fact has led to the adoption of HbA_1c_ as a diagnostic tool for diabetes by the International Expert Committee [1]. The question has now arisen as to whether HbA_1c_ measurement is useful in the assessment of macrovascular complications. Several population-based epidemiological studies in Asian populations [2–5] as well as in Western populations [6–13] have focused on the association between HbA_1c_ levels and intima-media thickness (IMT) of carotid arteries, which is generally accepted as a marker of the early stage of atherosclerosis. However, this issue has not been assessed sufficiently in a general Japanese population [5].

Glycated albumin (GA) and 1,5-anhydroglucitol (1,5-AG) levels, which are serum markers of short-term glycemic status, have also been shown to be intimately related to microvascular complications in recent epidemiological studies, including our previous report [14–16], and there has been a growing interest in GA and 1,5-AG as alternative markers of hyperglycemia [17]. Although several studies have examined the associations of GA [15, 18–25] and 1,5-AG [26, 27] levels with atherosclerosis among patients on dialysis and those with diabetes or hypertension, very few studies have been conducted to clarify this association in general populations [5, 28]. In addition, to our knowledge, there are no data comparing HbA_1c_, GA, 1,5-AG, fasting plasma glucose (FPG), and 2-hour postload glucose (PG) measurements as a tool for the assessment of atherosclerosis.

To investigate which glucose measure is more useful in the assessment of atherosclerosis, we examined the associations of HbA_1c_, GA, 1,5-AG, FPG, and 2-hour PG levels with carotid IMT in a community-dwelling Japanese population stratified by glucose tolerance status, and compared the magnitude of the influence of these five glycemic measures on the ability to discriminate carotid wall thickening.

## Methods

### Study population

A population-based prospective study of cardiovascular disease (CVD) and its risk factors has been underway since 1961 in the town of Hisayama, a suburb of the Fukuoka metropolitan area on Japan’s Kyushu Island. The population of the town has been stable for 50 years and was approximately 8400 in 2010. The age and occupational distributions, and nutritional intake of the population were similar to those of Japan as a whole based on data from the national census and nutrition survey [29]. In 2007 and 2008, a cross-sectional survey for the present study was performed in the town. A detailed description of this survey was published previously [16, 30]. Among a total of 3835 residents aged 40–79 years based on the town registry, 2957 (77.1 %) took part in a comprehensive assessment, including a 75-g oral glucose tolerance test (OGTT), the measurement of HbA_1c_, and a carotid ultrasound examination. We excluded eight subjects who did not consent to participate in the study, 46 who had already had breakfast, 35 who did not undergo the OGTT due to receiving insulin therapy, and 156 who refused the OGTT, leaving a total of 2712 subjects who completed the OGTT, HbA_1c_ measurement, and carotid ultrasound. Among these, 10 subjects without measurement of GA or 1,5-AG were excluded, and the remaining 2702 subjects (1198 men and 1504 women) were enrolled in the present study.

### Clinical evaluation and laboratory measurements

The study subjects underwent the OGTT between 8:00 and 10:30 A.M. after an overnight fast of at least 12 h. Blood for the glucose assay was obtained by venipuncture into tubes containing sodium fluoride at fasting and at 2-hour postload, and was separated into plasma and blood cells within 20 min. Plasma glucose concentrations were determined by the hexokinase method. Glucose tolerance status was classified as normal glucose tolerance (NGT) and glucose intolerance (GI) according to the 1998 World Health Organization criteria [31]; namely, for NGT, FPG <6.1 mmol/L and 2-hour PG <7.8 mmol/L; for GI, FPG ≥6.1 mmol/L, 2-hour PG ≥7.8 mmol/L, or the use of antidiabetic medications. Diabetes was defined as FPG ≥7.0 mmol/L, 2-hour PG ≥11.1 mmol/L, or the use of antidiabetic medications. HbA_1c_ levels were measured by latex aggregation immunoassay (Determiner HbA1C; Kyowa Medex, Tokyo, Japan). The values for HbA_1c_ were estimated as a National Glycohemoglobin Standardization Program (NGSP) equivalent value calculated with the formula: HbA_1c_ (%) = 1.02 × HbA_1c_ (Japan Diabetes Society) (%) + 0.25 % [32]. A portion of each serum specimen was stored at -80 °C for 5 years until it was used for measurement of GA and 1,5-AG in 2012. Serum GA levels were determined by an enzymatic method using an albumin-specific proteinase, ketoamine oxidase, and an albumin assay reagent (Lucica GA-L; Asahi Kasei Pharma, Tokyo, Japan). Serum 1,5-AG concentrations were measured enzymatically (Lana 1,5AG Auto Liquid; Nippon Kayaku, Tokyo, Japan). The intra- and interassay coefficients of variation were 0.6–1.1 % and 1.4–3.4 % for HbA_1c_, 0.5–3.2 % and 1.3 % for GA, 1.1–2.0 % and 2.5–2.8 % for 1,5-AG, and 0.4–1.3 % and 1.9–3.0 % for plasma glucose, respectively. Serum insulin values were measured by a chemiluminescent enzyme immunoassay. The homeostasis model assessment of insulin resistance (HOMA-IR) was calculated with the formula: FPG (mmol/L) × fasting serum insulin (μU/mL) / 22.5 [33], and subjects in the highest quartile of HOMA-IR distribution in our study population (HOMA-IR ≥2.07) were defined as having insulin resistance [31]. Serum total, low-density lipoprotein (LDL), and high-density lipoprotein (HDL) cholesterols and triglycerides were all determined enzymatically. Hyper-LDL cholesterolemia was defined as LDL cholesterol levels ≥3.62 mmol/L or the use of lipid-lowering medications [34]. Freshly voided urine samples were tested by the dipstick method. Proteinuria was defined as 1+ or more. Serum creatinine concentrations were measured enzymatically, and estimated glomerular filtration rate (eGFR) (mL/min/1.73 m^2^) was calculated using the following modified equations of the Chronic Kidney Disease Epidemiology Collaboration (CKD-EPI) for Japanese [35]: for men with a serum creatinine level >0.9 mg/dL, eGFR = 141 × (serum creatinine/0.9)^-1.209^ × 0.993^Age^ × 0.813; for men with a serum creatinine level ≤0.9 mg/dL, eGFR = 141 × (serum creatinine/0.9)^-0.411^ × 0.993^Age^ × 0.813; for women with a serum creatinine level >0.7 mg/dL, eGFR = 144 × (serum creatinine/0.7)^-1.209^ × 0.993^Age^ × 0.813; for women with a serum creatinine level ≤0.7 mg/dL, eGFR = 144 × (serum creatinine/0.7)^-0.329^ × 0.993^Age^ × 0.813. Chronic kidney disease (CKD) was defined as either an eGFR <60 mL/min/1.73 m^2^ or the presence of proteinuria [36].

The height and weight were measured with the subject in light clothes without shoes, and the body mass index (BMI) (kg/m^2^) was calculated. Blood pressure was obtained three times using an automated sphygmomanometer (BP-203 RVIIIB; Omron Healthcare Co., Ltd., Kyoto, Japan) with the subject in a sitting position after rest for at least 5 min; the average values were used in the analyses. Hypertension was defined as a systolic blood pressure ≥140 mmHg, a diastolic blood pressure ≥90 mmHg, or current treatment with antihypertensive agents.

Each participant completed a self-administered questionnaire covering medical history, antidiabetic, antihypertensive, and lipid-lowering treatments, alcohol intake, smoking habits, and physical activity. Alcohol intake and smoking habits were classified as either current use or not. Current smoking was defined as when the subject smoked at least 1 cigarette per day. Current drinking was defined as when the subject consumed at least 1 alcohol beverage per month. Subjects engaging in sports at least three times per week during their leisure time were defined as the regular exercise group. History of CVD was defined as any preexisting events of stroke or coronary heart disease, including myocardial infarction and coronary intervention. All cardiovascular events were adjudicated on the basis of physical examinations and a review of all available clinical information including medical records and imaging.

### Carotid ultrasonography

Carotid ultrasound was performed in a supine position using a real-time, B-mode ultrasound imaging unit (Toshiba Sonolayer SSA-250A; Toshiba, Tokyo, Japan) with a 7.5-MHz annular array probe. The ultrasound examination was carried out by specially trained laboratory technicians using a standardized technique. The technicians were blinded to the medical history, and clinical and laboratory data of each participant. The IMT was measured using the long-axis view of each common carotid artery. An image was obtained in the region 20 mm proximal to the origin of the bulb at the far wall of each common carotid artery. The IMT values at 250 computer-based points in the region were measured on each side using a computer-assisted measurement system (Intimascope; Media Cross Co., Ltd., Tokyo, Japan) [37]. The maximum IMT was defined as the largest IMT value in either the left or right common carotid artery, and carotid wall thickening was defined as a maximum IMT of >1.0 mm according to the Japan Academy of Neurosonology and the Japan Society of Ultrasonics in Medicine’s guidelines [38, 39].

### Statistical analysis

The SAS software package version 9.3 (SAS Institute, Cary, NC) was used to perform all statistical analyses. The differences in the mean values or frequencies of risk factors between glucose tolerance categories were assessed using the linear or logistic regression model, respectively. HbA_1c_, GA, 1,5-AG, FPG, and 2-hour PG levels were divided into quartiles by the presence or absence of GI. The values of IMT, fasting insulin, HOMA-IR, and triglycerides were transformed into logarithms to improve the skewed distribution, and geometric means were reported by back transformation. The linear regression model was used to examine the associations of 1 SD change in glycemic measures with the maximum IMT. The adjusted average values of the maximum IMT were calculated using the analysis of covariance method, and their trends across the quartiles of glycemic measures were tested by the linear regression model. The crude and multivariable-adjusted odds ratios (ORs) and their 95 % confidence intervals (CIs) for the presence of carotid wall thickening were estimated using the logistic regression model. Because serum 1,5-AG levels are decreased in the presence of hyperglycemia, the highest quartile was used as the reference group for 1,5-AG, while the lowest quartile was defined as the reference category for other glycemic measures. The heterogeneity in the influence of each glycemic measure on carotid wall thickening between subjects with and without other cardiovascular risk factors was assessed by adding an interaction term to the relevant statistical model. To compare the discrimination for the presence of carotid wall thickening between the models adjusted for known cardiovascular risk factors with and without continuous values of each glycemic measure, the difference in the area under the receiver operator characteristic curve (AUC) among models was estimated using the method of DeLong et al. [40]. A value of *p* < 0.05 was considered statistically significant in all analyses.

### Ethical considerations

This study was conducted with the approval of the Kyushu University Institutional Review Board for Clinical Research, and written informed consent was obtained from all the participants.

## Results

Of the study participants, there were 1603 (59.3 %) with NGT and 1099 (40.7 %) with GI. Among subjects with GI, 145 (13.2 %) had isolated fasting hyperglycemia (FPG ≥6.1 mmol/L and 2-hour PG <7.8 mmol/L), 457 (41.6 %) had isolated 2-hour postload hyperglycemia (FPG <6.1 mmol/L and 2-hour PG ≥7.8 mmol/L), and 497 (45.2 %) had both fasting and 2-hour postload hyperglycemia (FPG ≥6.1 mmol/L and 2-hour PG ≥7.8 mmol/L). The clinical characteristics of subjects are shown by GI status in Table 1. The mean values of age, maximum IMT, HbA_1c_, GA, FPG, 2-hour PG, fasting insulin, HOMA-IR, systolic and diastolic blood pressures, BMI, LDL cholesterol, and triglycerides, and the frequencies of men, insulin resistance, hypertension, antihypertensive and lipid-lowering medications, hyper-LDL cholesterolemia, proteinuria, CKD, and history of CVD were significantly higher in subjects with GI than in those with NGT, and subjects with GI had significantly lower 1,5-AG, HDL cholesterol, and eGFR values. The mean values of total cholesterol and the frequencies of alcohol intake, smoking habits, and regular exercise did not differ between the groups.

**Table 1:** Clinical characteristics of subjects, 2007–2008

	Total	Glucose tolerance status		
		Normal	Glucose intolerance	*P* value
	n = 2702	n = 1603	n = 1099	
Age (years)	60 (10)	58 (11)	63 (9)	<0.001
Men (%)	44.3	37.5	54.3	<0.001
Maximum IMT (mm)	0.97 (0.96 to 0.98)	0.94 (0.93 to 0.95)	1.01 (1.00 to 1.02)	<0.001
Hemoglobin A_1c_ (%)	5.5 (0.7)	5.2 (0.4)	5.9 (0.9)	<0.001
(mmol/mol)	37 (8)	33 (4)	41 (10)	<0.001
Glycated albumin (%)	15.2 (2.8)	14.4 (1.3)	16.4 (3.7)	<0.001
1,5-anhydroglucitol (μg/mL)	20.2 (8.3)	22.2 (7.4)	17.3 (8.7)	<0.001
Fasting plasma glucose (mmol/L)	5.8 (1.2)	5.3 (0.4)	6.5 (1.5)	<0.001
2-hour postload glucose (mmol/L)	8.0 (3.7)	6.0 (1.1)	10.8 (4.3)	<0.001
Fasting insulin (pmol/L)	39.3 (38.5 to 40.2)	34.6 (33.7 to 35.5)	47.3 (45.7 to 49.0)	<0.001
HOMA-IR	1.39 (1.36 to 1.42)	1.13 (1.10 to 1.16)	1.87 (1.80 to 1.94)	<0.001
Insulin resistance (%)	24.9	12.9	42.5	<0.001
Diabetes (%)	15.4	0	37.9	0.93
Antidiabetic medication (%)	6.4	0	15.7	0.93
Systolic blood pressure (mmHg)	131 (19)	126 (18)	138 (18)	<0.001
Diastolic blood pressure (mmHg)	80 (11)	77 (11)	83 (10)	<0.001
Hypertension (%)	45.6	33.0	64.0	<0.001
Antihypertensive medication (%)	28.5	19.0	42.4	<0.001
BMI (kg/m^2^)	23.2 (3.4)	22.5 (3.1)	24.3 (3.5)	<0.001
Total cholesterol (mmol/L)	5.45 (0.93)	5.44 (0.94)	5.47 (0.93)	0.42
LDL cholesterol (mmol/L)	3.24 (0.80)	3.21 (0.80)	3.29 (0.81)	0.02
HDL cholesterol (mmol/L)	1.73 (0.46)	1.81 (0.47)	1.62 (0.43)	<0.001
Triglycerides (mmol/L)	1.21 (1.18 to 1.23)	1.08 (1.06 to 1.11)	1.41 (1.36 to 1.46)	<0.001
Hyper-LDL cholesterolemia (%)	42.5	37.1	50.3	<0.001
Lipid-lowering medication (%)	14.3	9.5	21.3	<0.001
Current drinking (%)	51.9	51.2	52.9	0.38
Current smoking (%)	21.2	21.5	20.8	0.67
Regular exercise (%)	12.4	12.0	12.9	0.46
eGFR (mL/min/1.73 m^2^)	76.3 (10.9)	77.4 (10.4)	74.7 (11.5)	<0.001
Proteinuria (%)	5.0	2.9	8.2	<0.001
Chronic kidney disease (%)	10.8	7.8	15.1	<0.001
History of CVD (%)	4.4	2.8	6.6	<0.001

Maximum IMT, fasting insulin, HOMA-IR, and triglycerides values are shown by geometric means and 95 % confidence intervals due to the skewed distribution

All other values are given as the mean (SDs) or as a percentage

Insulin resistance was defined as HOMA-IR ≥2.07 (the highest quartile of HOMA-IR in total study population)

Diabetes was defined as fasting plasma glucose ≥7.0 mmol/L, 2-hour postload glucose ≥11.1 mmol/L, or the use of antidiabetic medications

Hypertension was defined as blood pressure ≥140/90 mmHg or current use of antihypertensive agents

Hyper-LDL cholesterolemia was defined as LDL cholesterol ≥3.62 mmol/L or the use of lipid-lowering medications

Proteinuria was defined as 1+ or more

Chronic kidney disease was defined as either an eGFR <60 mL/min/1.73 m^2^ or the presence of proteinuria

Regular exercise was defined as engaging in sports at least three times per week during leisure time

*IMT* intima-media thickness, *HOMA-IR* homeostasis model assessment of insulin resistance, *BMI* body mass index, *LDL* low-density lipoprotein, *HDL* high-density lipoprotein, *eGFR* estimated glomerular filtration rate, *CVD* cardiovascular disease

**Fig. 1:**
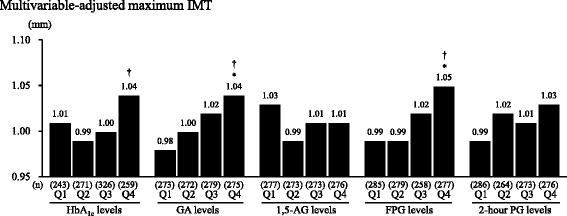
Multivariable-adjusted geometric average of maximum intima-media thickness according to quartiles of each glycemic measure in subjects with glucose intolerance. IMT: intima-media thickness; HbA_1c_: hemoglobin A_1c_; GA: glycated albumin; 1,5-AG: 1,5-anhydroglucitol; FPG: fasting plasma glucose; 2-hour PG: 2-hour postload glucose. * *p* < 0.01 vs Quartile 1, † p for trend <0.05. Multivariable adjustment was made for age, sex, hypertension, low-density lipoprotein cholesterol, high-density lipoprotein cholesterol, body mass index, alcohol intake, smoking habits, regular exercise, and lipid-lowering medication. HbA_1c_: Q1, <5.4 (36); Q2, 5.4–5.6 (36–38); Q3, 5.7–6.2 (39–44); Q4, ≥6.3 % (45 mmol/mol); GA: Q1, <14.3; Q2, 14.3–15.3; Q3, 15.4–17.1; Q4, ≥17.2 %; 1,5-AG: Q1, <11.0; Q2, 11.0–16.9; Q3, 17.0–22.9; Q4, ≥23.0 μg/mL; FPG: Q1, <5.7; Q2, 5.7–6.1; Q3, 6.2–6.8; Q4, ≥6.9 mmol/L; 2-hour PG: Q1, <8.0; Q2, 8.0–9.1; Q3, 9.2–12.3; Q4, ≥12.4 mmol/L

We estimated the crude and multivariable-adjusted geometric average of the maximum IMT according to the quartiles of each glycemic measure in subjects with GI. The crude geometric average of the maximum IMT increased significantly with rising HbA_1c_, GA, FPG, and 2-hour PG levels (all p for trend <0.001), but no clear association was observed for 1,5-AG levels (p for trend = 0.30). Similar associations were also observed in subjects with NGT (p for trend <0.05 for HbA_1c_, GA, FPG, and 2-hour PG; p for trend = 0.32 for 1,5-AG). Among subjects with GI, positive trends for HbA_1c_ (p for trend = 0.048), GA (p for trend <0.001), and FPG (p for trend <0.001), but not 2-hour PG (p for trend = 0.07), remained significant even after adjustment for cardiovascular risk factors; namely, age, sex, hypertension, LDL cholesterol, HDL cholesterol, BMI, alcohol intake, smoking habits, regular exercise, and lipid-lowering medication (Fig. 1). Meanwhile, in those with NGT, the significant associations between levels of any glycemic measures and the maximum IMT disappeared after adjustment for the above-mentioned confounding factors (Additional file 1: Figure S1).

In subjects with GI, when continuous variables of glycemic measures were used instead of quartile categories, HbA_1c_, GA, FPG, and 2-hour PG levels were significantly and positively associated with the maximum IMT even after adjustment for other cardiovascular factors, but no such association was seen for 1,5-AG (Table 2). The magnitude of the influence of 1 SD increment in HbA_1c_ (β = 0.021, *p* < 0.001), GA (β = 0.024, *p* < 0.001), and FPG (β = 0.024, *p* < 0.001) was larger than that of 2-hour PG (β = 0.014, *p* = 0.03). In those with NGT, no significant associations were found for all five glycemic measures after adjustment for other confounding factors (Additional file 1: Table S1).

**Table 2:** The associations of 1 SD increment in HbA_1c_, GA, FPG, and 2-hour PG, and 1 SD decrement in 1,5-AG with the maximum IMT in subjects with glucose intolerance

Glycemic measures	Crude		Multivariable-adjusted	
	β (95 % CI)	*P* value	β (95 % CI)	*P* value
HbA_1c_, per 0.9 % (10 mmol/mol) increment	0.028 (0.015 to 0.041)	<0.001	0.021 (0.009 to 0.034)	<0.001
GA, per 3.7 % increment	0.036 (0.023 to 0.049)	<0.001	0.024 (0.012 to 0.036)	<0.001
1,5-AG, per 8.7 μg/mL decrement	0.004 (-0.010 to 0.017)	0.59	0.003 (-0.009 to 0.015)	0.61
FPG, per 1.5 mmol/L increment	0.030 (0.017 to 0.043)	<0.001	0.024 (0.011 to 0.036)	<0.001
2-hour PG, per 4.3 mmol/L increment	0.029 (0.016 to 0.043)	<0.001	0.014 (0.002 to 0.026)	0.03

Multivariable adjustment was made for age, sex, hypertension, low-density lipoprotein cholesterol, high-density lipoprotein cholesterol, body mass index, alcohol intake, smoking habits, regular exercise, and lipid-lowering medication

*HbA*_*1c*_ hemoglobin A_1c_, *GA* glycated albumin, *1,5-AG* 1,5-anhydroglucitol, *FPG* fasting plasma glucose, *2-hour PG* 2-hour postload glucose, *CI* confidence interval

The crude and multivariable-adjusted ORs and their 95 % CIs for the presence of carotid wall thickening according to the quartiles of each glycemic measure in subjects with GI are shown in Table 3. The crude ORs for the presence of carotid wall thickening significantly increased with elevating HbA_1c_, GA, FPG, and 2-hour PG levels, while lower 1,5-AG levels were significantly associated with prevalent carotid wall thickening. These associations remained robust even after adjustment for other confounding factors for HbA_1c_, GA, and FPG (all p for trend <0.001), but not for 1,5-AG (*p* = 0.09) and 2-hour PG (*p* = 0.14). In subjects with NGT, although the crude OR for carotid wall thickening did not increase with decreasing 1,5-AG levels, HbA_1c_, GA, FPG, and 2-hour PG levels were significantly and positively associated with the presence of carotid wall thickening (Additional file 1: Table S2). However, these upward trends were markedly attenuated and no longer significant after multivariable adjustment.

**Table 3:** Crude and multivariable-adjusted ORs and 95 % CIs for the presence of carotid wall thickening according to quartiles of glycemic measures in subjects with glucose intolerance

	No. of cases/subjects	Crude OR (95 % CI)	*P* value	P for trend	Multivariable-adjusted OR (95 % CI)	*P* value	P for trend
HbA_1c_, % (mmol/mol)							
Q1, <5.4 (36)	99/243	1.00 (reference)		<0.001	1.00 (reference)		<0.001
Q2, 5.4–5.6 (36–38)	107/271	0.95 (0.67 to 1.35)	0.77		0.94 (0.64 to 1.37)	0.73	
Q3, 5.7–6.2 (39–44)	168/326	1.55 (1.11 to 2.16)	0.01		1.32 (0.91 to 1.92)	0.15	
Q4, ≥6.3 (45)	156/259	2.20 (1.54 to 3.15)	<0.001		1.96 (1.32 to 2.93)	<0.001	
GA, %							
Q1, <14.3	102/273	1.00 (reference)		<0.001	1.00 (reference)		<0.001
Q2, 14.3–15.3	114/272	1.21 (0.86 to 1.71)	0.28		1.23 (0.84 to 1.79)	0.29	
Q3, 15.4–17.1	146/279	1.84 (1.31 to 2.58)	<0.001		1.68 (1.15 to 2.45)	0.007	
Q4, ≥17.2	168/275	2.63 (1.87 to 3.72)	<0.001		2.12 (1.45 to 3.10)	<0.001	
1,5-AG, μg/mL							
Q1, <11.0	158/277	1.47 (1.05 to 2.06)	0.02	0.03	1.43 (0.99 to 2.07)	0.05	0.09
Q2, 11.0–16.9	120/273	0.87 (0.62 to 1.22)	0.41		0.87 (0.60 to 1.26)	0.47	
Q3, 17.0–22.9	121/273	0.88 (0.63 to 1.23)	0.46		0.97 (0.67 to 1.41)	0.88	
Q4, ≥23.0	131/276	1.00 (reference)			1.00 (reference)		
FPG, mmol/L							
Q1, <5.7	121/285	1.00 (reference)		<0.001	1.00 (reference)		<0.001
Q2, 5.7–6.1	120/279	1.02 (0.73 to 1.43)	0.89		0.86 (0.59 to 1.23)	0.40	
Q3, 6.2–6.8	123/258	1.24 (0.88 to 1.73)	0.22		1.26 (0.87 to 1.82)	0.23	
Q4, ≥6.9	166/277	2.03 (1.45 to 2.84)	<0.001		1.76 (1.21 to 2.56)	0.003	
2-hour PG, mmol/L							
Q1, <8.0	119/286	1.00 (reference)		<0.001	1.00 (reference)		0.14
Q2, 8.0–9.1	123/264	1.22 (0.87 to 1.72)	0.24		1.04 (0.72 to 1.50)	0.85	
Q3, 9.2–12.3	125/273	1.19 (0.85 to 1.66)	0.32		0.92 (0.64 to 1.32)	0.64	
Q4, ≥12.4	163/276	2.02 (1.45 to 2.83)	<0.001		1.41 (0.97 to 2.04)	0.07	

Multivariable adjustment was made for age, sex, hypertension, low-density lipoprotein cholesterol, high-density lipoprotein cholesterol, body mass index, alcohol intake, smoking habits, regular exercise, and lipid-lowering medication. Because serum 1,5-AG levels are decreased in the presence of hyperglycemia, the highest quartile was used as the reference group for 1,5-AG

*HbA*_*1c*_ hemoglobin A_1c_, *GA* glycated albumin, *1,5-AG* 1,5-anhydroglucitol, *FPG* fasting plasma glucose, *2-hour PG* 2-hour postload glucose, *OR* odds ratio, *CI* confidence interval

We next performed stratified analyses to examine whether the associations of glycemic measures with the presence of carotid wall thickening differed between groups with and without other cardiovascular risk factors in subjects with GI (Additional file 1: Figure S2A and S2B). Overall, there were no statistically significant differences in the multivariable-adjusted ORs for the prevalent carotid wall thickening per 1 SD increment in HbA_1c_, GA, FPG, and 2-hour PG, and 1 SD decrement in 1,5-AG according to levels of other risk factors: sex, age (<65 and ≥65 years), smoking habits, insulin resistance, hypertension, and hyper-LDL cholesterolemia (all p for heterogeneity >0.05).

To evaluate the improvement in discriminative ability for detecting the presence of carotid wall thickening by addition of continuous values of each glycemic measure to the model including potential cardiovascular risk factors, we compared the AUC between the models with and without each glycemic measure among subjects with GI (Table 4). The AUC increased significantly by adding HbA_1c_ (0.729, *p* = 0.04) or GA (0.728, *p* = 0.04) values to the model including the potential cardiovascular risk factors mentioned above (0.719). When 1,5-AG (0.721, *p* = 0.27), FPG (0.724, *p* = 0.19), and 2-hour PG (0.721, *p* = 0.39) values were added, the AUC did not increase significantly.

**Table 4:** Comparison of the discriminative ability for detecting the presence of carotid wall thickening between the models with and without each glycemic measure among subjects with glucose intolerance

Model	Area under curve (95 % CI)	*P* value (vs. Model 1)
Model 1	0.719 (0.689 to 0.748)	reference
Model 1 + HbA_1c_	0.729 (0.699 to 0.758)	0.04
Model 1 + GA	0.728 (0.699 to 0.758)	0.04
Model 1 + 1,5-AG	0.721 (0.691 to 0.751)	0.27
Model 1 + FPG	0.724 (0.694 to 0.753)	0.19
Model 1 + 2-hour PG	0.721 (0.691 to 0.751)	0.39

Model 1 includes age, sex, hypertension, low-density lipoprotein cholesterol, high-density lipoprotein cholesterol, body mass index, alcohol intake, smoking habits, regular exercise, and lipid-lowering medication

*HbA*_*1c*_ hemoglobin A_1c_, *GA* glycated albumin, *1,5-AG* 1,5-anhydroglucitol, *FPG* fasting plasma glucose, *2-hour PG* 2-hour postload glucose

We also performed sensitivity analyses excluding subjects with history of CVD and found that the findings were not substantially altered (data not shown).

## Discussion

Using data from a cross-sectional survey in a Japanese community, we demonstrated that in subjects with GI, elevated levels of HbA_1c_, GA, and FPG were more strongly associated with increased maximum IMT compared to 1,5-AG and 2-hour PG, independent of other cardiovascular risk factors. Similar results were obtained from analysis of the associations between glycemic measures and the presence of carotid wall thickening. There were no clear differences in the influence of each glycemic measure between subjects with and without other cardiovascular risk factors. Furthermore, adding HbA_1c_ or GA to a model including known cardiovascular risk factors significantly improved the assessment of the likelihood of prevalent carotid wall thickening in those with GI. These findings suggest that HbA_1c_ and GA provide superior discrimination for the presence of carotid wall thickening, which is an early stage of atherosclerosis, compared to other glycemic measures in community-dwelling Japanese subjects with GI.

In the present study, increased HbA_1c_ levels were significantly and positively associated with the presence of carotid wall thickening in individuals with GI, independently of other cardiovascular risk factors. Other Asian population studies have also shown that higher HbA_1c_ levels were significantly and independently related to increased carotid IMT in Chinese populations [2, 3] and an Asian Indian population [4]. Similar findings were observed in most studies in Western populations [6, 8–10, 13]. With regard to clinical CVD events, although some cohort studies in the U.S. have demonstrated that HbA_1c_ levels were not independently predictive of CVD incidence and mortality [41, 42], our previous study revealed that elevated HbA_1c_ levels were significantly associated with increased risk of the development of CVD [43]. Other prospective studies in Western populations have also shown significant positive associations between HbA_1c_ levels and the risk of CVD [9, 44]. These findings suggest that a higher HbA_1c_ level is a risk factor for subclinical and clinical CVD in Asian populations including Japanese as well as in Western populations.

Epidemiological studies of community-dwelling persons which investigated the association between GA levels and atherosclerosis have been scarce. Only a Japanese community-based study has reported that maximum carotid IMT increased in subjects with higher GA levels, although it did not fully take confounding factors into consideration [5]. In our study, the likelihood of carotid wall thickening increased linearly with elevating GA levels among individuals with GI, and this association remained robust even after controlling for other cardiovascular risk factors. With regard to clinical studies, a recent case-cohort study in subjects with type 1 diabetes failed to demonstrate a significant influence of GA levels on CVD incidence and death [15]. Another study of patients with type 1 diabetes has also reported no significant association between GA/HbA_1c_ ratio and carotid IMT [25]. However, other studies of patients on dialysis and those with type 2 diabetes have shown that higher GA levels were associated with the presence and progression of carotid atherosclerosis [20, 22, 24], the presence of coronary artery disease [18], impaired coronary collateral growth [23], and future CVD mortality [21] or hospitalization [19]. These findings, when taken together with our present results, indicate a close association between GA levels and the risk of atherosclerosis.

The present analysis demonstrated no clear association of 1,5-AG levels with maximum carotid IMT even in the crude analysis. Prior clinical studies also did not reveal significant differences in mean or maximum carotid IMT across 1,5-AG levels in patients with diabetes or hypertension [26, 27]. These findings imply that a weak association exists between 1,5-AG levels and carotid atherosclerosis. One of the possible reasons for this phenomenon is that the 1,5-AG level depends on the magnitude of glycosuria rather than plasma glucose levels [17], while other glycemic measures, including HbA_1c_, GA, FPG and 2-hour PG, directly reflect the degree of hyperglycemia. Another reason may be that 1,5-AG levels were affected by individual differences in the renal thresholds for glucose [45]. These explanations may account for the relatively weak association between 1,5-AG levels and carotid atherosclerosis in our study. On the other hand, with regard to clinical CVD events, one population-based cohort study in Japan has shown that lower 1,5-AG levels significantly increased the risk of developing CVD in men [28]. Since few studies have investigated the associations between 1,5-AG levels and the risk of CVD, further research is needed to clarify this issue.

In our study, higher FPG levels were independently related to carotid wall thickening in subjects with GI, but 2-hour PG levels were not. There have been conflicting data regarding the association between 2-hour PG levels and carotid atherosclerosis. At least 4 studies have shown that higher 2-hour PG levels were not a relevant factor for carotid atherosclerosis [3, 7, 11, 46], although others have found that elevated 2-hour PG levels were associated with increased carotid IMT [6, 47, 48]. Our previous prospective study showed that increased 2-hour PG levels were an independent risk factor for the occurrence of clinical CVD [49]. Thus, 2-hour PG levels might be more associated with the advanced stages of atherosclerosis than the early stages. Further investigation is necessary to validate this hypothesis.

In the present study, adding HbA_1c_ or GA to a model including known cardiovascular risk factors significantly increased the AUCs for the presence of carotid wall thickening in subjects with GI, while the addition of 1,5-AG, FPG, and 2-hour PG did not substantially increase them. These results suggest that HbA_1c_ and GA provide greater improvement in discriminative ability for detecting carotid atherosclerosis than other glycemic measures. Advanced glycation end products (AGEs) are recognized as one of the important contributors to the pathogenesis of atherosclerosis in hyperglycemia [50]. It is also known that HbA_1c_ and GA are non-enzymatically glycated proteins, and these two measures are regarded as precursors of AGEs [51, 52]. Thus, it is speculated that HbA_1c_ and GA values are correlated with the amount of AGEs. These findings raise the possibility that AGEs may play a key role in the early stages of atherosclerosis. Furthermore, it has been reported that HbA_1c_ had a strong correlation with elevated FPG levels, which are mainly caused by insulin resistance, while GA was closely related to higher postprandial glucose levels, which are attributed to reduced insulin secretion [53]. Considering this finding, higher HbA_1c_ levels may indicate insulin resistance, while elevated GA levels are likely to reflect decreased insulin secretion, resulting in postprandial glucose excursion. Since insulin resistance [54], abdominal obesity [55, 56], and glucose excursion [57] were also found to play important roles in the pathogenesis of atherosclerosis, higher HbA_1c_ and GA levels may contribute to increased carotid IMT through insulin resistance, glucose excursion as well as AGEs, and this might be the reason why measurements of HbA_1c_ and GA improve the discriminative ability for the presence of carotid wall thickening. In addition, because HbA_1c_ and GA measurements can be done without fasting or timed samples, these two measures are convenient and suitable for use in general practice. This advantage has implications for the identification and management of atherosclerosis in its early stages, and thus measurements of HbA_1c_ and GA suggest to be useful in the assessment of atherosclerosis.

In our subjects with NGT, the crude ORs for the presence of carotid wall thickening increased with rising HbA_1c_, GA, FPG, and 2-hour PG levels, but these associations were markedly attenuated after adjustment for other covariates. These findings suggest that higher glucose levels within normal range may not contribute independently to increased carotid IMT. Since other known cardiovascular risk factors, such as aging, hypertension, and dyslipidemia, tended to accumulate at the higher levels of HbA_1c_, GA, FPG, and 2-hour PG in our subjects with NGT (data not shown), individuals with elevations of these glycemic measures would seem to have higher carotid IMT through the mediation of other coexisting risk factors.

The strengths of our study include the population-based design and high participation rate. We also made a comparison of the five glycemic measures, which was not done in prior studies. However, some limitations should be mentioned. First, the cross-sectional study design limits the interpretation of causality between levels of glycemic measures and carotid atherosclerosis. Since the usefulness of glycemic measures in the assessment of atherosclerosis would ideally be evaluated in studies that examine the association between measures of glycemia and incident atherosclerotic disease, further prospective investigation is expected. Second, each glycemic measure was based on a single measurement, as was the case in most other epidemiological studies. This limitation may have resulted in misclassification of study subjects into different categories, and such misclassification could have weakened the association found in our study, biasing the results toward the null hypothesis. Third, the serum GA and 1,5-AG levels were measured after being stored at -80 °C for 5 years. In this context, however, we should note that the stability of GA and 1,5-AG measurements in frozen stored serum sample has been confirmed [58, 59]. Fourth, several laboratory technicians took ultrasound images of carotid artery without assessment of inter-observer variability. However, they were specially trained to use a standardized technique, and the IMT was measured automatically using a computer-assisted measurement system. Thus, we believe that this limitation is not likely to invalidate the findings observed in the present study. Fifth, CKD may cause a change in 1,5-AG levels through alterations in renal hemodynamics. However, the sensitivity analyses excluding subjects with CKD did not make any material difference in the findings on the association between 1,5-AG levels and carotid IMT (data not shown). Lastly, sample size of our study was relatively small to perform analyses separately in subjects with diabetes and prediabetes. Further studies with larger sample size are required to elucidate this issue.

## Conclusions

The present analysis showed that, in a Japanese population with GI, elevated HbA_1c_, GA, and FPG levels were significantly associated with increased carotid IMT, and that the ability of HbA_1c_ and GA to detect carotid wall thickening was superior to that of 1,5-AG, FPG, and 2-hour PG, suggesting that measurements of HbA_1c_ and GA are useful as markers of early atherosclerosis. Further prospective studies are needed to verify these findings.

## Additional file

Additional file 1: Figure S1. Multivariable-adjusted geometric average of maximum intima-media thickness according to quartiles of each glycemic measure in subjects with normal glucose tolerance. **Table S1:** The associations of 1 SD increment in HbA_1c_, GA, FPG, and 2-hour PG, and 1 SD decrement in 1,5-AG with the maximum intima-media thickness in subjects with normal glucose tolerance. **Table S2:** Crude and multivariable-adjusted odds ratios and 95 % confidence intervals for the presence of carotid wall thickening according to quartiles of glycemic measures in subjects with normal glucose tolerance. **Figure S2A and S2B:** Multivariable-adjusted odds ratios and their 95 % confidence intervals for the presence of carotid wall thickening per 1 SD increment in HbA_1c_, GA, FPG, and 2-hour PG, and 1 SD decrement in 1,5-AG according to the subgroups of sex, age, smoking habits, insulin resistance, hypertension, and hyper-LDL cholesterolemia in subjects with glucose intolerance.

## Abbreviations

HbA_1c_: Hemoglobin A_1c_
IMT: Intima-media thickness
GA: Glycated albumin
1,5-AG: 1,5-anhydroglucitol
FPG: Fasting plasma glucose
PG: Postload glucose
CVD: Cardiovascular disease
OGTT: Oral glucose tolerance test
NGT: Normal glucose tolerance
GI: Glucose intolerance
NGSP: National Glycohemoglobin Standardization Program
HOMA-IR: Homeostasis model assessment of insulin resistance
LDL: Low-density lipoprotein
HDL: High-density lipoprotein
eGFR: Estimated glomerular filtration rate
CKD-EPI: Chronic Kidney Disease Epidemiology Collaboration
CKD: Chronic kidney disease
BMI: Body mass index
OR: Odds ratio
CI: Confidence interval
AUC: Area under the receiver operating characteristic curve
AGE: Advanced glycation end product
