# Supplementary material for: Association of hemoglobin A1c and glycated albumin with carotid atherosclerosis in community-dwelling Japanese subjects: the Hisayama Study
Source: Cardiovasc Diabetol. 2015 Jun 24;14:84. doi: 10.1186/s12933-015-0247-7 (PMC4482030; doi:10.1186/s12933-015-0247-7)
Supplement: Additional file 1: Figure S1. — Multivariable-adjusted geometric average of maximum intima-media thickness according to quartiles of each glycemic measure in subjects with normal glucose tolerance. Table S1: The associations of 1 SD increment in HbA1c, GA, FPG, and 2-hour PG, and 1 SD decrement in 1,5-AG with the maximum intima-media thickness in subjects with normal glucose tolerance. Table S2: Crude and multivariable-adjusted odds ratios and 95 % confidence intervals for the presence of carotid wall thickening according to quartiles of glycemic measures in subjects with normal glucose tolerance. Figure S2A and S2B: Multivariable-adjusted odds ratios and their 95 % confidence intervals for the presence of carotid wall thickening per 1 SD increment in HbA1c, GA, FPG, and 2-hour PG, and 1 SD decrement in 1,5-AG according to the subgroups of sex, age, smoking habits, insulin resistance, hypertension, and hyper-LDL cholesterolemia in subjects with glucose intolerance. [file 12933_2015_247_MOESM1_ESM.docx]

**Supplemental Data**

**Title: Association of hemoglobin A_1c_ and glycated albumin with carotid atherosclerosis in community-dwelling Japanese subjects: the Hisayama Study**

**Authors:**

Naoko Mukai, Toshiharu Ninomiya, Jun Hata, Yoichiro Hirakawa, Fumie Ikeda,

Masayo Fukuhara, Taeko Hotta, Masafumi Koga, Udai Nakamura, Dongchon Kang,

Takanari Kitazono, and Yutaka Kiyohara

**Supplemental Figure Legend**

Supplemental Figure S1: Multivariable-adjusted geometric average of maximum intima-media thickness according to quartiles of each glycemic measure in subjects with normal glucose tolerance.

IMT: intima-media thickness; HbA_1c_: hemoglobin A_1c_; GA: glycated albumin; 1,5-AG: 1,5-anhydroglucitol; FPG: fasting plasma glucose; 2-hour PG: 2-hour postload glucose

Multivariable adjustment was made for age, sex, hypertension, low-density lipoprotein cholesterol, high-density lipoprotein cholesterol, body mass index, alcohol intake, smoking habits, regular exercise, and lipid-lowering medication.

HbA_1c_: Q1, <5.0 (31); Q2, 5.0-5.1 (31-32); Q3, 5.2-5.4 (33-36); Q4, ≥5.5% (37 mmol/mol); GA: Q1, <13.7; Q2, 13.7-14.3; Q3, 14.4-15.2; Q4, ≥15.3%; 1,5-AG: Q1, <17.2; Q2, 17.2-21.6; Q3, 21.7-26.6; Q4, ≥26.7 μg/mL; FPG: Q1, <5.1; Q2, 5.1-5.2; Q3, 5.3-5.5; Q4, ≥5.6 mmol/L; 2-hour PG: Q1, <5.3; Q2, 5.3-6.1; Q3, 6.2-6.8; Q4, ≥6.9 mmol/L.

Supplemental Figure S2A and S2B: Multivariable-adjusted odds ratios and their 95% confidence intervals for the presence of carotid wall thickening per 1 SD increment in HbA_1c_, GA, FPG, and 2-hour PG, and 1 SD decrement in 1,5-AG according to the subgroups of sex, age, smoking habits, insulin resistance, hypertension, and hyper-LDL cholesterolemia in subjects with glucose intolerance.

HbA_1c_: hemoglobin A_1c_; GA: glycated albumin; 1,5-AG: 1,5-anhydroglucitol; FPG: fasting plasma glucose; 2-hour PG: 2-hour postload glucose; OR: odds ratio; CI: confidence interval; IR: insulin resistance; HT: hypertension; High LDL: hyper-LDL cholesterolemia

Multivariable adjustment was made for age, sex, hypertension, low-density lipoprotein cholesterol, high-density lipoprotein cholesterol, body mass index, alcohol intake, smoking habits, regular exercise, and lipid-lowering medication. The variable relevant to the subgroup was excluded from each model.

Diamonds indicate point estimates of odds ratios, and bars indicate 95% confidence intervals.

:

Supplemental Table S1. The associations of 1 SD increment in HbA_1c_, GA, FPG, and 2-hour PG, and 1 SD decrement in 1,5-AG

with the maximum IMT in subjects with normal glucose tolerance

| Glycemic measures | Crude | |  | Multivariable-adjusted | |
| --- | --- | --- | --- | --- | --- |
|  | β (95% CI) | P value |  | β (95% CI) | P value |
| HbA_1c_, per 0.4% (4 mmol/mol) increment | 0.023 (0.012 to 0.034) | <0.001 |  | -0.007 (-0.017 to 0.003) | 0.16 |
|  |  |  |  |  |  |
| GA, per 1.3% increment | 0.017 (0.006 to 0.028) | 0.003 |  | -0.009 (-0.021 to 0.002) | 0.10 |
|  |  |  |  |  |  |
| 1,5-AG, per 7.4 μg/mL decrement | -0.001 (-0.012 to 0.010) | 0.85 |  | 0.001 (-0.009 to 0.011) | 0.81 |
|  |  |  |  |  |  |
| FPG, per 0.4 mmol/L increment | 0.033 (0.022 to 0.044) | <0.001 |  | 0.008 (-0.002 to 0.018) | 0.12 |
|  |  |  |  |  |  |
| 2-hour PG, per 1.1 mmol/L increment | 0.020 (0.009 to 0.031) | <0.001 |  | -0.006 (-0.017 to 0.004) | 0.21 |

HbA_1c_: hemoglobin A_1c_; GA: glycated albumin; 1,5-AG: 1,5-anhydroglucitol; FPG: fasting plasma glucose; 2-hour PG: 2-hour

postload glucose; CI: confidence interval.

Multivariable adjustment was made for age, sex, hypertension, low-density lipoprotein cholesterol, high-density lipoprotein

cholesterol, body mass index, alcohol intake, smoking habits, regular exercise, and lipid-lowering medication.

Supplemental Table S2. Crude and multivariable-adjusted ORs and 95% CIs for the presence of carotid wall thickening according to

Supplemental Figure S1

quartiles of glycemic measures in subjects with normal glucose tolerance

|  | No. of | Crude | P value | P for trend |  | Multivariable-adjusted | P value | P for trend |
| --- | --- | --- | --- | --- | --- | --- | --- | --- |
|  | cases/subjects | OR (95% CI) |  |  |  | OR (95% CI) |  |  |
| HbA_1c_, % (mmol/mol) |  |  |  |  |  |  |  |  |
| Q1, <5.0 (31) | 106/365 | 1.00 (reference) |  | <0.001 |  | 1.00 (reference) |  | 0.63 |
| Q2, 5.0-5.1 (31-32) | 116/382 | 1.07 (0.78 to 1.46) | 0.69 |  |  | 0.83 (0.58 to 1.19) | 0.31 |  |
| Q3, 5.2-5.4 (33-36) | 132/385 | 1.28 (0.94 to 1.74) | 0.12 |  |  | 0.88 (0.62 to 1.25) | 0.47 |  |
| Q4, ≥5.5 (37) | 188/471 | 1.62 (1.21 to 2.17) | 0.001 |  |  | 0.88 (0.63 to 1.24) | 0.47 |  |
|  |  |  |  |  |  |  |  |  |
| GA, % |  |  |  |  |  |  |  |  |
| Q1, <13.7 | 134/417 | 1.00 (reference) |  | 0.02 |  | 1.00 (reference) |  | 0.27 |
| Q2, 13.7-14.3 | 109/376 | 0.86 (0.64 to 1.17) | 0.34 |  |  | 0.77 (0.55 to 1.09) | 0.14 |  |
| Q3, 14.4-15.2 | 154/429 | 1.18 (0.89 to 1.57) | 0.25 |  |  | 0.95 (0.67 to 1.34) | 0.77 |  |
| Q4, ≥15.3 | 145/381 | 1.30 (0.97 to 1.74) | 0.08 |  |  | 0.74 (0.51 to 1.08) | 0.12 |  |
|  |  |  |  |  |  |  |  |  |
| 1,5-AG, μg/mL |  |  |  |  |  |  |  |  |
| Q1, <17.2 | 141/400 | 1.14 (0.85 to 1.53) | 0.37 | 0.19 |  | 1.13 (0.80 to 1.58) | 0.49 | 0.26 |
| Q2, 17.2-21.6 | 145/400 | 1.19 (0.89 to 1.60) | 0.23 |  |  | 1.32 (0.95 to 1.84) | 0.10 |  |
| Q3, 21.7-26.6 | 126/400 | 0.97 (0.72 to 1.30) | 0.82 |  |  | 1.02 (0.73 to 1.42) | 0.90 |  |
| Q4, ≥26.7 | 130/403 | 1.00 (reference) |  |  |  | 1.00 (reference) |  |  |
|  |  |  |  |  |  |  |  |  |
| FPG, mmol/L |  |  |  |  |  |  |  |  |
| Q1, <5.1 | 108/390 | 1.00 (reference) |  | <0.001 |  | 1.00 (reference) |  | 0.08 |
| Q2, 5.1-5.2 | 125/423 | 1.10 (0.81 to 1.49) | 0.56 |  |  | 0.85 (0.60 to 1.19) | 0.34 |  |
| Q3, 5.3-5.5 | 140/404 | 1.39 (1.02 to 1.87) | 0.03 |  |  | 0.95 (0.68 to 1.34) | 0.78 |  |
| Q4, ≥5.6 | 169/386 | 2.03 (1.51 to 2.74) | <0.001 |  |  | 1.30 (0.92 to 1.83) | 0.14 |  |
|  |  |  |  |  |  |  |  |  |
| 2-hour PG, mmol/L |  |  |  |  |  |  |  |  |
| Q1, <5.3 | 117/397 | 1.00 (reference) |  | 0.009 |  | 1.00 (reference) |  | 0.09 |
| Q2, 5.3-6.1 | 130/391 | 1.19 (0.88 to 1.61) | 0.25 |  |  | 0.88 (0.62 to 1.24) | 0.45 |  |
| Q3, 6.2-6.8 | 145/426 | 1.24 (0.92 to 1.66) | 0.16 |  |  | 0.79 (0.56 to 1.10) | 0.17 |  |
| Q4, ≥6.9 | 150/389 | 1.50 (1.12 to 2.02) | 0.007 |  |  | 0.76 (0.54 to 1.07) | 0.11 |  |

HbA_1c_: hemoglobin A_1c_; GA: glycated albumin; 1,5-AG: 1,5-anhydroglucitol; FPG: fasting plasma glucose; 2-hour PG: 2-hour postload

glucose; OR: odds ratio; CI: confidence interval.

Multivariable adjustment was made for age, sex, hypertension, low-density lipoprotein cholesterol, high-density lipoprotein cholesterol,

body mass index, alcohol intake, smoking habits, regular exercise, and lipid-lower medication.

Because serum 1,5-AG levels are decreased in the presence of hyperglycemia, the highest quartile was used as the reference group for 1,5-AG.
